# What Emotion Facial Expressions Tell Us About the Health of Others

**DOI:** 10.3389/fpsyg.2020.585242

**Published:** 2020-11-13

**Authors:** Shlomo Hareli, Or David, Ursula Hess

**Affiliations:** ^1^The Interdisciplinary Center for Research on Emotions, School of Business Administration, University of Haifa, Haifa, Israel; ^2^Department of Psychology, Humboldt University of Berlin, Berlin, Germany

**Keywords:** age stereotypes, facial expressions, COVID-19, health perception, emotions

## Abstract

To avoid contagion, we need information about the health status of those whom we engage with. This is especially important when we have cause for concern that the other is indeed sick, such as is the case during the world-wide outbreak of the coronavirus in 2020. In three studies, one conducted several years before the pandemic, and two during the pandemic, we showed that facial expressions of emotions are used as signals of health status. Specifically, happy expressers are perceived as healthier than expressers showing negative emotions or neutrality (Studies 1–3), whereas anger was interpreted as a signal of ill health (Study 3). Importantly, however, facial expressions affected health perception only when there was a prior reason to suspect ill health. This was the case for older expressers before and after the pandemic for whom age-related stereotypes set expectations of ill health and for all ages during a wide-spread pandemic, which extends this suspicion to everyone. In Study 3, we showed that the effect of emotion expressions was also generalized to the physical distance that the observer wishes to keep from the expresser. Overall, this research is the first to show a role of emotion expressions in informing health perception.

## Introduction

Information about the health of others is crucial for avoiding contagious diseases (Neuberg et al., 2011). Whereas this is always true, it becomes even more relevant when there is a pandemic such as the world-wide outbreak of the coronavirus which started in late 2019. One way to deal with the risk of catching a contagious disease via contact with others is physical distancing. That is, reducing the number of close social contacts that people make (Williams et al., 2015). Yet, keeping everyone at a distance for a long period of time is practically impossible. The lives of humans are heavily based on interacting with many other people, including strangers, for different purposes of varying importance. Accordingly, people may need to decide with whom contact should be avoided and who is safe. That is, they need to assess whether others pose a health risk to them because other people may be sick and hence contagious. This raises the question of what to base such an assessment on especially if that person is a stranger encountered accidentally.

Several appearance cues can be used as signals of health. These include the color and texture of the face (Jones et al., 2004; Fink et al., 2006a,b; Feinberg, 2008) or the body shape of a person (Hill and Silver, 1995). Lesions, disfigurements, and other morphological abnormalities can also indicate illness (Faulkner et al., 2004) as can other changes such as in the sound of the person’s voice (Feinberg, 2008). These cues serve as signals of ill health because they reflect an anomaly from what is expected to be the normal or healthy state of a person and hence may be a direct derivative of some illness. Yet, it is not necessarily the case that all contagious diseases, for everyone during all stages of the disease, involve changes that are readily detectable by others. Other, more transient, cues about the person’s state such as those reflected by a person’s emotions may also be taken as signals of a person’s health. In line with this idea, we tested the possibility that the emotional expressions of others are perceived by their observers as a source of such information.

Further, we assume that the degree to which emotion expressions are taken as signals of a person’s health varies as a function of the perceived likelihood that the other is sick, which is related to the identity of the other person. That is, certain individuals are perceived as more at risk than others. Specifically, people may be more inclined to presume that people that belong to certain social groups, such as old people, are more likely to be sick than younger people (McTavish, 1971; Montepare and Zebrowitz-McArthur, 1988; Gluth et al., 2010). The same is true of unfamiliar foreigners (Faulkner et al., 2004). Yet, unfamiliar foreigners are more likely to be seen as a source of a contagious disease when a person is aware of the existence of a disease in the population (Orbell et al., 2015).

We report the results of three studies that explored how the expression of different emotions by young and old expressers (Studies 1 and 2) and young people from three different ethnic groups, one of these expected to be less familiar to participants (Study 3), affected the assessments of the expressers’ health by others. The first study was conducted several years before the start of the COVID-19 virus outbreak in 2019 (in 2014) and the other two during the outbreak (April, 2020). This enabled us to assess whether the extent to which an observers’ use of the emotions of others as signals of health varies as a function of the prevalence of high risk during a pandemic. In what follows, we describe the rationale for our research question.

### Facial Expressions as Signals of Health

As suggested above, knowledge about the health of others is presumed to be crucial for avoiding contagious diseases (Neuberg et al., 2011). The notion that emotion expressions signal health status is based on appraisal theory (e.g., Frijda, 1986). Specifically, emotions arise when something important for the emoter occurs and the specific emotion felt reflects how the emoter appraises the situation (Frijda, 1986). According to appraisal theories of emotions, (e.g., Scherer, 1987) when something desirable to the individual occurs, a positive emotion is a more likely reaction. By contrast, when something undesirable occurs, the expected emotion is a negative one. The specific positive or negative emotion experienced will further depend on appraisals of additional aspects of the situation. For example, when an undesirable situation is appraised to be caused by something that the emoter sees as in their power to handle, anger is the likely response. By contrast, when the undesirable situation seems to be caused by something that the emoter cannot handle, sadness is the more likely response (Scherer, 1987). People have a naïve grasp of what causes specific emotions and often use this knowledge to infer how the emoter perceives the environment (Hareli and Hess, 2010).

Accordingly, when a person expresses a positive emotion, the observer can infer that nothing negative bothers the expresser. Since, by definition, someone who is unhealthy would be less likely to feel well, one can conversely infer that the expresser of a positive emotion is in good health. In fact, we often refer to being sick as being unwell. By contrast, when a person expresses a negative emotion, the observer can infer that something bad happened to the emoter. However, there are many potential causes for a negative emotion, hence the conclusion that the person is unwell requires additional situational information (see below). Also, even within such a situational context, not all negative emotions are likely to be seen as signaling bad health to the same degree. As mentioned above, anger, unlike sadness, reflects a situation perceived by the emoter as one they can handle (Scherer, 1987). If one can handle a negative situation, the situation is likely to be less severe than one that cannot be handled. Thus, sadness should be a more likely signal of bad health than anger.

Nevertheless, as noted above, in order for a negative emotion to be perceived as signaling the health of a person, it should be attributed to one’s health condition. Contexts that make this attribution likely are those where the observer may already suspect ill health. One factor that may affect the likelihood for such a suspicion is the age of the person.

Age stereotypes tend to depict older people as ill, tired, less active, and less capable of adapting to changes (McTavish, 1971; Montepare and Zebrowitz-McArthur, 1988; Gluth et al., 2010). Accordingly, one may be more inclined to attribute negative emotions to health issues in the case of older people than younger ones.

Another factor that may cause people to be more attentive to cues that reflect the health of another is the general health situation. Thus, during an outbreak of an infectious disease, people would be more attentive to illness cues than at other times. This expectation is in line with findings indicating that people are inclined to think more about illness when illness is more prevalent than when it is not, such as when there is a flu outbreak (Orbell et al., 2015).

Finally, past research has shown that people who feel more vulnerable to a contagious disease are likely to react especially negatively toward members of unfamiliar outgroups (Faulkner et al., 2004). In line with this finding, one may expect observers to be inclined to pay more attention to illness cues when the person encountered is a member of an unfamiliar outgroup than a member of the in-group or a familiar outgroup. As such, as a pandemic makes sickness more salient, people are more likely to feel vulnerable to a contagious disease and should therefore be more attentive to sickness cues in unfamiliar others. This expectation is in line with the idea that evolved mechanisms such as that of avoiding a contagious disease are responsive to contextual variables (Schaller, 2003).

To test these ideas, we report the results of three studies. In the first two, participants saw a picture of a young or old man or woman expressing either, happiness, anger, sadness, or neutrality. Participants were asked to assess how healthy this person seemed to them. The first study was conducted in the summer of 2014 as part of a different project and has not been published. The second study was a replication of the first study conducted during the COVID-19 pandemic (April, 2020). The last study, which was also conducted during the COVID-19 pandemic, used only photos of young people who were either of a Northern European origin, Middle Eastern origin, or of an Eastern European origin. The data can be downloaded upon request from OSF. The research was conducted in accordance with the ethical standards of the American Psychological Association and the local IRB.

## Study 1

The goal of Study 1 was to assess whether expressions of emotions inform health perception and the degree to which they do so as a function of expressers’ age group which may reflect their susceptibility to being sick.

### Method

#### Participants

A total of 561 (318 men) participants with a mean age of 36 years (SD = 11.87, range: 18–86) were recruited via Amazon Mechanical Turk, completed the study, and passed tests of due diligence. These tests included two checks. First, following the instructions, they were asked about the type of stimuli they would see. The second attention check consisted of a slider ranging from 0 – “Not at all” to 6 – “To a large degree” and participants were asked to set the slider to the “Not at all” option. Participants, who did not read the instructions or who click answers without reading the questions were thus excluded.

Based on a sensitivity analysis using G^∗^Power (Faul et al., 2007), given our sample size, the minimum effect size that the experiment had 80% power to detect was *f* = 0.14.

#### Stimuli

Facial expressions of happiness, anger, and sadness, as well as a neutral expression from four male and four female expressers for each age group (age < 30 and age > 70) were taken from the Montreal Set of Facial Displays of Emotion (MSFDE; Beaupré and Hess, 2005). Importantly, a directed facial action task was used to assure that expressions were equivalent both in regard to action units and intensity. In this study as well as the others, each participant saw only one expresser who was a young or old man or woman, showing one of the above mentioned emotional expressions.

#### Dependent Measures

Participants were instructed to provide their first impressions of the person shown in the photo with regard to their health as well as weight, height, age, and IQ and likeability. The additional ratings were included to disguise our interest in health specifically and the question about perceived health was the sixth question. Health ratings were made on a 7-point scale anchored with 0-not at all healthy and 6- very healthy.

### Results and Discussion

A 2 (age group: young vs. old) × 2 (expresser gender) × 4 (expressed emotion: anger, sadness, happiness, and neutrality) between-subjects analysis of variance was conducted on ratings of perceived health. The expected main effects of age group, *F*(1,545) = 46.62, *p* < 0.001, η_p_^2^ = 0.08, and emotion expression, *F*(3,545) = 6.45, *p* < 0.001, η_p_^2^ = 0.03, emerged significantly. This effect was qualified by a two-way interaction between age-group and emotion expression, *F*(3,545) = 2.64, *p* = 0.049, η_p_^2^ = 0.01. *Post hoc* analyses (*p* < 0.05) revealed that, for young expressers, the emotions shown did not influence perceptions of health (M_happiness_ = 4.03, SD = 1.15, CI95%: 3.77; 4.29, M_neutrality_ = 4.00, SD = 0.96, CI95%: 3.76; 4.24, M_anger_ = 3.71, SD = 1.25, CI95%: 3.42; 4.01; and, M_sadness_ = 3.70, SD = 1.12, CI95%: 3.42; 3.98).

By contrast, a happy older person (*M* = 3.71, SD = 0.98, CI95%: 3.47; 3.96) was rated as healthier than an older person expressing any other emotion, which did not differ in terms of perceived health (M_sadness_ = 3.05, SD = 1.19, CI95%: 2.80; 3.31, M_neutrality_ = 3.03, SD = 1.14, CI95%: 2.75; 3.31, and, M_anger_ = 3.13, SD = 1.24, CI95%: 2.87; 3.39).

Further, young expressers were perceived as healthier than older expressers, for the negative emotions and neutrality. However, when older expressers showed happiness they were perceived as equally healthy as young expressers (on whose perceived health emotion did not have an effect).

Overall, health perception was influenced by emotion expression for older expressers but not for younger ones. The finding that happy older individuals were perceived as healthy as were the younger individuals is consistent with the notion that the stereotypical expectation for older people is one of unhealthiness. As such, when older people express happiness, signaling that all is well, this is taken as a sign of good health.

## Study 2

In Study 1, we found evidence that when observers have a reason to suspect that someone is not well, the emotions that this person expresses can signal the health status of a person. Specifically, because age-related stereotypes (McTavish, 1971; Montepare and Zebrowitz-McArthur, 1988; Gluth et al., 2010) create a default expectation that old people are sick, expressions of happiness are taken as individuating information that overrides this default impression and thus leads to the assumption that this person is healthy. And, in fact, not any less healthy then a young person. Yet, age is not the only type of information that can cause people to think about the health of others. When there is an outbreak of a pandemic, awareness of the fact that people may be sick may also increase the tendency of observers to attribute the emotion expressions of others to their health. In Study 2, we tested this possibility by replicating Study 1 during the height of the COVID-19 pandemic (April 2020).

### Method

#### Participants

A total of 483 (244 men) participants with a mean age of 41 years (SD = 13.31, range: 18–76) were recruited through Amazon Mechanical Turk, completed the study, and passed tests of due diligence. Based on a sensitivity analysis using G^∗^Power (Faul et al., 2007), given our sample size, the minimum effect size that the experiment had 80% power to detect was *f* = 0.15.

#### Procedure

The same stimuli and dependent measures as used in Study 1 were used. To verify that participants were aware of the COVID-19 pandemic, we added two additional questions at the end of the study on a separate page. Using the same answer format as used for the other questions, participants were first asked to report the extent to which, in their opinion, the existence of the COVID-19 pandemic affected them. Finally, they were asked if they or someone they knew was or had been sick with COVID-19 as a yes/no question.

### Results and Discussion

First, we assessed the degree to which participants reported being affected by the existence of the COVID-19 pandemic. To this aim, we conducted a single sample *t*-test against 0. This test revealed that participants reported being affected by the pandemic to a significant degree (*M* = 3.62, SD = 1.71, CI95% [3.46; 3.77]), *t*(482) = 46.41, *p* < 0.001, *d* = 2.12. Yet, the majority of the participants (77.4%) reported that neither they nor someone they know was or had been sick with COVID-19.

To assess whether emotion expressions affected the judgments of the participants on the health of the targets, we again conducted a 2 (age group: young vs. old) × 2 (expresser gender) × 4 (expressed emotion: anger, sadness, happiness, and neutrality) between-subjects analysis of variance on ratings of perceived health. As in Study 1, main effects of age group, *F*(1,467) = 77.86, *p* < 0.001, η_p_^2^ = 0.14, and emotion expression, *F*(3,467) = 8.43, *p* < 0.001, η_p_^2^ = 0.05, emerged significantly. As in Study 1, and as may be expected, younger targets were perceived as healthier (*M* = 4.13, SD = 1.23, CI95% [3.97; 4.27]) than older targets (*M* = 3.18, SD = 1.17, CI95% [3.01; 3.32]). *Post hoc* tests (*p* < 0.05) on the main effect of emotion expression, revealed that targets showing happiness seemed healthier (*M* = 4.09, SD = 1.25, CI95% [3.88; 4.28]) than targets showing any of the other expressions, which were rated similarly (M_neutrality_ = 3.54, SD = 1.25, CI95% [3.38; 3.81], M_anger_ = 3.60, SD = 1.24, CI95% [3.30; 3.73], M_sadness_ = 3.41, SD = 1.33, CI95% [3.16; 3.59]). No other effect emerged significantly.

We then conducted an exploratory general linear model analysis with OLS using the gamlj suite in Jamovi on the combined data for Study 1 (conducted before the COVID-19 pandemic) and Study 2 (conducted during the COVID-19 pandemic). We further included age of the participant as a covariate. Age of the participant was included because arguably older participants might have a different risk perception.

The 2 (time period) × 2 (target age group) × 2 (target gender) × 4 (emotion) × participant age analysis yielded a significant main effect of participant age, *F*(1,993) = 6.81, *p* = 0.009, η_p_^2^ = 0.01, such that older participants rated everyone at both times as healthier (*r* = 0.07, *p* = 0.032), but no significant interaction with any of the other factors (all *p* > 0.077).

In addition, significant main effects of emotion, *F*(3,993) = 14.53, *p* < 0.001, η_p_^2^ = 0.04, and target age group, *F*(1,993) = 118.15, *p* < 0.001, η_p_^2^ = 0.11, were qualified by the significant expected emotion × target age group × time period interaction, *F*(3,993) = 2.63, *p* = 0.049, η_p_^2^ = 0.01. Specifically, simple effects analyses showed that, as suggested by the separate analyses, for the data collected before the pandemic (Study 1), the contrast between happiness and neutral expression was significant for the older target faces, β = 0.77, SE = 0.20, *t*(993) = 3.88, *p* < 0.001, but not for the younger target faces, β = 0.05, SE = 0.19, *t*(993) = 0.26, *p* = 0.798. By contrast, for the data collected during the pandemic, the contrast between happiness and neutral was significant for both the young, β = 0.55, SE = 0.22, *t*(993) = 2.53, *p* = 0.012 and the old target group, β = 0.44, SE = 0.20, *t*(993) = 2.16, *p* = 0.031. No further significant interactions emerged.

As such, this analysis confirms the difference in findings between Study 1 and Study 2. However, it should be noted that we did not manipulate time period, and as such, the data is only correlational. Further, the studies were not set up to be adequately powered for higher order interactions, which might have qualified the result.

We did not find any interaction between age of observer and age of expresser or any other factor. The main effect of participant age suggests that older participants rated everyone healthier, a finding that may be in line with the generally more positive outlook of older people (Carstensen and Mikels, 2005).

Overall, in both studies, expressions of happiness were taken as a signal that the expresser was healthy. However, in Study 1, this was only true for older expressers, whereas in Study 2 it was the case for both older and younger expressers.

In other words, when people have a reason to suspect the health status of someone, happiness expressions become a marker of good health. In Study 1, the suspicion of ill health was based on age stereotypes. In Study 2, however, the pandemic made the health status of everyone questionable (Orbell et al., 2015). Hence, expressions of happiness were taken as a signal of good health regardless of the age group of the expresser. In other words, expressions of happiness are seen as a signal that reduces the suspicion that someone is sick, be it due to the social identity of that person (i.e., old age) or the situation (i.e., the existence of a pandemic).

## Study 3

Both Studies 1 and 2 indicated that expressions of happiness can serve as signals that a person is healthy when there is a reason to suspect that the person is in fact not in good health. This was the case for older people due to age-related stereotypes (Study 1) or for all people during a pandemic (Study 2).

Yet, there is evidence that when people feel more vulnerable to a contagious disease, as is the case during a pandemic, they are likely to react especially negatively toward members of unfamiliar outgroups (Faulkner et al., 2004) and immigrants (Huang et al., 2011). In line with these findings, one may expect observers to be inclined to rely on emotion expressions as a signal of health status to a larger degree when confronted with a member of an unfamiliar outgroup than with a member of the in-group or a familiar outgroup (Faulkner et al., 2004; Huang et al., 2011).

To test this hypothesis, and to replicate the findings from Study 2 with regard to younger people, in Study 3, we used photos of posers from three distinct social groups, Northern Europe, Eastern Europe, and the Middle East. We expected that Middle-Easterners would be perceived as an unfamiliar out-group and hence their emotions, during the outbreak of the COVID-19 pandemic, would be taken as a signal of their health to a larger degree than would be the case for the other two groups. Finally, we also added a measure of the physical distance desired to be kept from the expresser, to get an additional measure of the possible effect of emotion expressions and social group on assessments of the health of the target.

### Method

#### Participants

A total of 730 (379 women, 1 other) participants with a mean age of 39 years (SD = 12.68, range: 19–76) were recruited through Amazon Mechanical Turk, completed the study, and passed tests of due diligence. Based on a sensitivity analysis using G^∗^Power (Faul et al., 2007), given our sample size, the minimum effect size that the experiment had 80% power to detect was *f* = 0.14.

#### Stimuli

Facial expressions of happiness, anger, and sadness, as well as a neutral expression from four male and four female expressers for the Northern European and Middle-Eastern groups were taken from the Amsterdam Dynamic Facial Expression Set (Van der Schalk et al., 2011) and the photos for the Eastern European group were taken from the Warsaw Set of Emotional Facial Expression Pictures (Olszanowski et al., 2015) that was developed with the help of the team that developed the Amsterdam set. Under each the photo of each person appeared a name typical of the respective social group to increase the chances that the expresser will be seen as belonging to the desired social group. The Northern European woman was named Elizabeth, the Eastern European woman was named Danka and the Middle-Eastern woman, Fahima. The Northern European man was named James, the Eastern European man was named Bogdan and the Middle-Eastern man, Ahmad.

#### Dependent Measures

The same dependent measures used in Study 2, were also used in this study with the addition of a measure of physical distance. Specifically, participants were asked to indicate the distance that they would keep from the other person. For this, the photo was shown again and two circles were drawn below it. One circle was labeled “you” and the other “other.” Participants were asked to click a Minus and Plus button to move the circles closer or further away from one another. The default distance was 6 ft and circles could be placed as close as 1 ft or at a maximum distance of 11 ft. Participants were also asked to report their ethnicity and the state they live in. This was done to assess the degree to which Middle Easterners can be expected to be unfamiliar.

### Results and Discussion

We assumed that Middle Easterners would constitute an unfamiliar group for our participants. Indeed, only one participant reported being of an Arab ethnicity, 70.7% considered themselves as Caucasian, 11% as Asian or pacific islanders, 9.3 as black and the rest as Hispanic, Indigenous or Aboriginal, Latino, or other. Participants were from 45 different states in the United States. The highest number of participants (10%) were from Florida, 9.3% from California, 9% from Texas, 6.3% from New York, and 6% or less were from other states. According to a report from the Migration Policy Institute Migration Policy Institute (2018) (January 10, 2018), in 2016, nearly 1.2 million immigrants from the Middle East lived in the United States. Slightly more than half of immigrants from the Middle East reside in five states: California (20%), Michigan (10%), New York (9%), and Texas and New Jersey (about 6% each). Together these statistics make it unlikely that more than very few of our participants encounter people from the Middle East frequently enough to consider them a familiar outgroup.

Next, as in Study 2, we assessed the degree to which participants reported being affected by the COVID-19 pandemic. To this aim, we conducted a single sample *t*-test against 0. Participants reported being significantly affected by the pandemic (*M* = 3.89, SD = 1.85, CI95% [3.76; 4.03]), *t*(729) = 56.94, *p* < 0.001, *d* = 2.10. As in Study 2, the majority of the participants reported that neither they nor someone they know was or had been sick with COVID-19 (81.10%).

To test the extent to which emotion expressions affected the judgment of participants of the targets’ health as a function of the expresser’s social group, we conducted a general liner model analysis with the three factors (social group: Northern Europe, Eastern Europe vs. the Middle-East) × 2 (expresser gender) × 4 (expressed emotion: anger, sadness, happiness, and neutrality), and participant age using the gamlj suite in jamovi. Only the main effects of participant age *F*(3,688) = 15.11, *p* < 0.001, η_p_^2^ = 0.02, emotion *F*(3,688) = 14.32, *p* < 001, η_p_^2^ = 0.06, and target gender *F*(3,688) = 4.88, *p* < 0.001, η_p_^2^ = 0.01, emerged significantly. As for Studies 1 and 2, older participants rated everyone healthier (*r* = 0.16, *p* < 0.001).

*Post hoc* tests revealed that, as in Study 2, expressers who showed happiness appeared healthier (*M* = 4.59, SD = 1.12, CI_95__%_ [4.41; 4.77]) than expressers who showed any of the other expressions. However, unlike in Studies 1 and 2, anger decreased the perceived health of the expresser (*M* = 3.75, SD = 1.34, CI_95__%_ [3.57; 3.93]) relative to happiness and neutrality (*M* = 4.24, SD = 1.20, CI_95__%_ [4.06; 4.41]), but not sadness (*M* = 4.09, SD = 1.27, CI_95__%_[3.91; 4.27]). With regard to gender, women (*M* = 4.27, SD = 1.25, CI_95__%_ [4.14; 4.40]) were rated as healthier than men (*M* = 4.07, SD = 1.28, CI_95__%_ 3.94; 4.19). No other effect emerged as significant.

To test the extent to which emotion expressions affected participants’ intentions to keep physical distance from the targets, general liner model analysis with the three factors (social group: Northern Europe, Eastern Europe vs. the Middle-East) × 2 (expresser gender) × 4 (expressed emotion: anger, sadness, happiness, and neutrality) and participant age. As for perceived health, a main effect of emotion expression, *F*(3,688) = 13.04, *p* < 0.001, η_p_^2^ = 0.05, and target gender, *F*(1,688) = 5.43, *p* = 0.020, η_p_^2^ = 0.01, emerged significantly. However, no significant main effect or interaction involving participant age emerged.

*Post hoc* tests (*p* < 0.05) revealed that participants reported wanting to be closer to happy expressers (*M* = 5.51, SD = 2.46, CI_95__%_ [5.13; 5.89]) than to neutral (*M* = 6.17, SD = 2.59, CI_95__%_ [5.79; 6.54]) and angry (*M* = 7.16, SD = 2.69, CI_95__%_ [6.78; 7.54]) expressers but not sad expressers (*M* = 5.91, SD = 2.57, CI_95__%_ [5.52; 6.30]). Sad and neutral expressers were rated similarly in terms of desired distance from the expresser. Notably, participants wished to keep a greater distance from an angry expresser than from expressers showing any other emotion. The main effect of gender matched the pattern of results for perceived health as participants wished to keep a greater distance from men (*M* = 6.41; SD = 2.65, CI_95__%_ [6.15; 6.68]) than from women (*M* = 5.96; SD = 2.62, CI_95__%_ [5.69; 6.23]).

Overall, as in Studies 1 and 2, expressions of happiness were taken as a signal that the expressers were healthy, yet this effect was not affected by the expressers’ social identity. In addition, unlike in Studies 1 and 2, angry expressers were perceived as less healthy than expressers showing any other emotion.

It is possible that this is due to the different expressions used in this study. Whereas in Study 1 and 2 images were black and white, in Study 3 we used colored photos. Nevertheless, we expected anger, if anything, to be seen as a signal of better health than sadness. This because anger, as opposed to sadness, reflects a situation perceived by the emoter as one they can handle (Scherer, 1987). If one can handle a negative situation, the situation is likely to be less severe than one they cannot handle. Our finding in this study, goes against this prediction. Yet, anger, first and foremost, reflects an undesirable situation and also one that is unjust (Frijda, 1986). One might speculate that falling ill can be seen as something bad and undeserved and in that sense, anger might be considered a sign of ill health.

That participants wanted to keep more distance from angry expressers matches the notion that anger signals ill health. However, since anger reflects intentions to attack, to move against another person or more generally, remove an obstruction, whereas happiness signals a desire to affiliate with others (Frijda, 1986; Frijda et al., 1989; Lazarus, 1991), we cannot exclude the possibility that ratings of desired distance reflect this aspect of the expression as well. That gender also had an effect such that perceivers thought that women are healthier than men and wished to be closer to them, may reflect the fact that news about COVID-19 reported men to be more susceptible to the disease than women (Ducharme, 2020, April 24), as such they may have felt that women posed less danger.

Notably social group did not affect ratings of perceived health. It is possible that newspaper reports that focused on the fact that the pandemic was evident across the globe, made everyone equally suspect of being potentially infected.

## General Discussion

Being able to assess whether another person is healthy or not is crucial for avoiding contagious diseases (Neuberg et al., 2011). This may become especially important when there is a pandemic such as the world-wide outbreak of the coronavirus late in 2019. Indeed, people are rather attentive to the health of others and use different cues related to the appearance of others as signals of their health (Faulkner et al., 2004; Jones et al., 2004; Fink et al., 2006a,b; Feinberg, 2008). In three different studies, one prior to the COVID-19 pandemic and two during it, we tested the assumption that expressions of emotions by strangers can be used by observers as cues to the health of these people. Further, we assumed that such cues are more likely to be used by observers for this goal when there is a reason to suspect that the expresser is unhealthy.

This may be the case when the person is older since age-related stereotypes suggest that old people are more likely to be sick (McTavish, 1971; Montepare and Zebrowitz-McArthur, 1988; Gluth et al., 2010). This is also likely the case when there is a widespread outbreak of a pandemic.

Overall, in three studies we were able to garner evidence in support of this notion. First, for older expressers prior to the pandemic and for all expressers during it, happiness signaled that the person was in good health. In addition, in Study 3 only, expressions of anger signaled poorer health.

That emotion expressions serve as cues to the health of people is consistent with the idea that people hold a naïve understanding of what causes different emotions (Weiner, 1987; Hareli and Hess, 2010). Based on this understanding, observers may infer potential causes for an emotion – in this case, the expressers’ health. Thus, knowing that positive emotions (e.g., happiness) reflect that the person’s situation is desirable and that negative emotions (e.g., anger) reflect that the person’s situation is undesirable, observers may use this information as a cue to health.

Yet, emotion expressions are most often not related to health. Hence, it is more likely that they are perceived as a sign of health when there is a reason to suspect that a person is not healthy. In our research there were two such reasons, first, stereotypes of the elderly suggest that older people are likely not healthy (McTavish, 1971; Montepare and Zebrowitz-McArthur, 1988; Gluth et al., 2010). Second, the prevailing pandemic made the potential for ill health salient.

Overall, the results of the present research, for the first time, show the potential of emotion expressions to signal the health of expressers. Further, they also indicate that whether emotion expressions are used in this way varies with situational factors such as the existence of a pandemic and/or the identity of the expresser that may suggest a potential for ill health. These perceptions also extend to an expressed desire to keep more distance from expressers who are perceived as less healthy as shown in Study 3.

We further compared findings from Studies 1 and 3 directly and this analysis conformed to the pattern of results found in the separate analyses. However, this analysis remains correlational as we did not manipulate the presence of the disease. Hence, it is still important for future research to directly test the effect of such an awareness. For example, by manipulating perceived prevalence of a disease.

Another aspect of importance in the present paper concerns the age of participants. Specifically, it is reasonable to assume that people for whom catching a disease has harsher consequences, as would be the case for most older people, will be more likely to be sensitive to cues reflecting health than others. Yet, it seems that older people see themselves, as well as others, at lower risk of catching COVID-19 than younger people do (Gerhold, 2020). We included participant age in our analyses as a covariate. We found that in all studies, older participants rated everyone healthier. This may be a further reflection of the notion that older participants perceive less risk of disease, or a general reflection of the more positive outlook of older people (Carstensen and Mikels, 2005).

Finally, it is important to test the effect of expressions or verbal reports of a wider range of emotions. The present findings are congruent with a valence-based account – positive emotions signal good health and negative emotions ill health. Yet, it is possible that certain negative emotions indicate better health and certain positive emotions poor health. For example, envy may be taken as a signal of good health because observers may assume that if the other person can afford feeling envious, probably nothing worse bothers them. Likewise, gratitude may be seen as a sign of weakness and hence may be reflecting some level of illness. Either way, emotion expressions carry the potential of informing observers about the health of expressers.

## Data Availability Statement

The datasets presented in this study can be found in online repositories. The names of the repository/repositories and accession number(s) can be found below: https://osf.io/yt9u7/?view_only=dcdfabae073c4305a162f92b8a944476.

## Ethics Statement

Ethical review and approval was not required for the study on human participants in accordance with the local legislation and institutional requirements. The patients/participants provided their written informed consent to participate in this study.

## Author Contributions

SH together with UH designed the study and wrote the manuscript. OD helped with literary review. SH together with OD programmed the studies and prepared the materials. All authors joined together to analyze the data.

## Conflict of Interest

The authors declare that the research was conducted in the absence of any commercial or financial relationships that could be construed as a potential conflict of interest.
